# Plasma prostasin: a novel risk marker for incidence of diabetes and cancer mortality

**DOI:** 10.1007/s00125-022-05771-w

**Published:** 2022-08-04

**Authors:** Xue Bao, Biao Xu, Iram Faqir Muhammad, Peter M. Nilsson, Jan Nilsson, Gunnar Engström

**Affiliations:** 1 Department of Cardiology, Nanjing Drum Tower Hospital, The Affiliated Hospital of Nanjing University Medical School, Nanjing, China; 2 Department of Clinical Sciences, Malmö, Lund University, Malmö, Sweden

**Keywords:** Cancer mortality, Cohort study, Diabetes, Prostasin

## Abstract

**Aims/hypothesis:**

Diabetes is associated with an increased risk of cancer. Prostasin is an epithelial sodium channel stimulator that has been associated with suppression of tumours, glucose metabolism and hyperglycaemia-associated tumour pathology. However, the association between prostasin, diabetes and cancer mortality has not been well investigated in humans. We aim to investigate the associations between plasma prostasin and diabetes, and to explore whether prostasin has an effect on cancer mortality risk in individuals with hyperglycaemia.

**Methods:**

Plasma prostasin was measured using samples from the Malmö Diet and Cancer Study Cardiovascular Cohort, and statistical analysis was performed from both sex-specific quartiles and per 1 SD. The cross-sectional association between plasma prostasin and diabetes was first studied in 4658 participants (age 57.5 ± 5.9 years, 39.9% men). After excluding 361 with prevalent diabetes, the associations of prostasin with incident diabetes and cancer mortality risk were assessed using Cox regression analysis. The interactions between prostasin and blood glucose levels as well as other covariates were tested.

**Results:**

The adjusted OR for prevalent diabetes in the 4th vs 1st quartile of prostasin concentrations was 1.95 (95% CI 1.39, 2.76) (*p* for trend <0.0001). During mean follow-up periods of 21.9 ± 7.0 and 23.5 ± 6.1 years, respectively, 702 participants developed diabetes and 651 died from cancer. Prostasin was significantly associated with the incidence of diabetes. The adjusted HR for diabetes in the 4th vs 1st quartile of prostasin concentrations was 1.76 (95% CI 1.41, 2.19) (*p* for trend <0.0001). Prostasin was also associated with cancer mortality There was a significant interaction between prostasin and fasting blood glucose for cancer mortality risk (*p* for interaction =0.022), with a stronger association observed in individuals with impaired fasting blood glucose levels at baseline (HR per 1 SD change 1.52; 95% CI 1.07, 2.16; *p*=0.019).

**Conclusions/interpretation:**

Plasma prostasin levels are positively associated with diabetes risk and with cancer mortality risk, especially in individuals with high blood glucose levels, which may shed new light on the relationship between diabetes and cancer.

**Graphical abstract:**

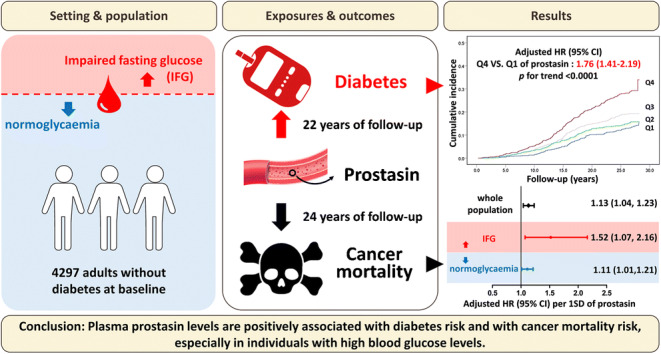

**Supplementary Information:**

The online version of this article (10.1007/s00125-022-05771-w) contains peer-reviewed but unedited supplementary material.

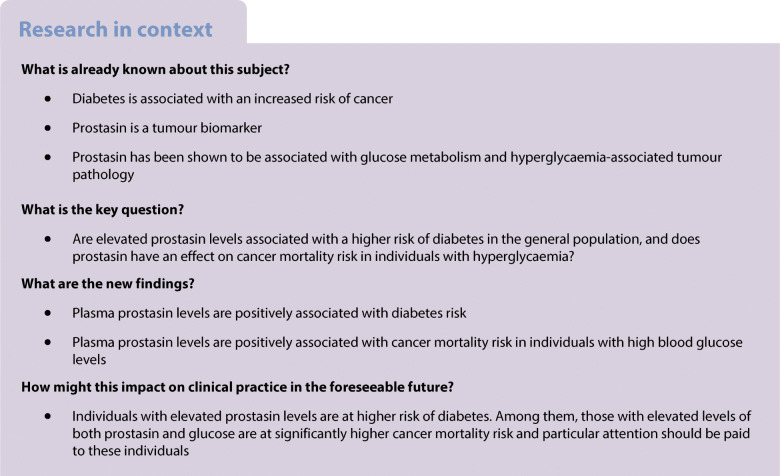



## Introduction

Prostasin, also known as protease serine S1 family member 8, is an extracellular serine protease with a trypsin-like cleavage specificity [1]. It is a well-known stimulator of epithelial sodium channels (ENaCs) that is widely expressed in epithelial tissues [1]. ENaCs are pivotal regulators of sodium balance, and are composed of three homologous subunits (α, β and γ) [2]. Prostasin facilitates full activation of ENaCs by activating cleavage of the γ subunits [1, 3]. Inhibiting prostasin is a potential mechanism for treatment of ENaC activation-related diseases such as cystic fibrosis and hypertension [1, 4, 5].

In addition to regulating ENaCs, accumulating evidence from expression, cell culture and animal studies supports a suppressive effect of prostasin on tumours at various sites including head, neck and oral squamous cells, breast, colorectum and prostate [6, 7]. Consequently, decreased prostasin expression is associated with poor cancer outcomes [7, 8]. However, ovarian carcinoma appears to be an exception, whereby prostasin is overexpressed in ovaries [9, 10], and elevated serum prostasin concentration serves as a potential diagnostic marker for ovarian carcinoma [10, 11]. There is extensive evidence showing that diabetes is associated with an increased risk of cancer and cancer mortality [12], and the association may be altered to some extent by glucose-lowering treatments [13]. The biological connection between these two conditions is still obscure, but several possible mechanisms have been proposed [13]. Among them, hyperglycaemia-induced epithelial-to-mesenchymal transition is considered to facilitate cancer metastasis [14]. The ability to inhibit the epithelial-to-mesenchymal transition in cancer cells is an important explanation for prostasin-dependent tumour suppression [6, 7, 15]. This suggests the possibility of a suppressive effect of prostasin against hyperglycaemia-induced tumours.

Current evidence regarding the role of prostasin in the development of diabetes is limited. Two experimental studies have reported a protective effect of hepatic prostasin against glucose and lipid metabolism dysfunction [16, 17]. In addition, ENaCs and insulin share many similarities in terms of biogenesis [18]. Insulin has long been recognised as a powerful stimulator of ENaCs [19], and recent evidence also suggests a regulatory effect of ENaCs on cell viability and insulin content in pancreatic beta cells [20]. It is thus speculated that an association may also exist between prostasin and diabetes.

To our knowledge, no epidemiological study has yet investigated the association of prostasin levels with diabetes or cancer mortality in humans. In this study, based on a large Swedish population, we aim to investigate the cross-sectional and longitudinal associations between plasma prostasin levels and diabetes, as well as the association between prostasin and cancer mortality. We also explore whether prostasin and its interaction with baseline hyperglycaemia have an effect on cancer mortality risk.

## Methods

### Study population

The Malmö Diet and Cancer Study is a large population-based prospective study from Malmö, a city in southern Sweden [21]. During 1991–1994, a random sample (*n*=6103) of participants from the Malmö Diet and Cancer Study were invited to participate in a sub-cohort study, the Malmö Diet and Cancer Study Cardiovascular Cohort [22]. Of these, 4658 participants had complete data on prostasin and covariates, and the cross-sectional association between prostasin and diabetes was assessed in these participants (age 57.5 ± 5.9 years, 39.9% men) (Electronic supplementary material (ESM) Fig. 1). We then excluded 361 participants with prevalent diabetes, defined as self-reported diabetes or use of glucose-lowering medication or fasting venous blood glucose concentration ≥6.1 mmol/l (corresponding to a fasting plasma glucose concentration cut-off of 7.0 mmol/l [23]). Therefore, 4297 participants remained for the prospective analysis (age 57.3 ± 5.9 years, 38.5% men), including 232 with a history of cancer and 93 with cardiovascular diseases at baseline. Information on insulin levels was available for 4627 and 4247 participants in the cross-sectional and prospective analyses, respectively.

All participants provided written informed consent. The study conformed to the ethical guidelines of the 1975 Declaration of Helsinki, and was approved by the Lund University Ethical Committee (LU51/90, LU 2009/633, LU 2011/537 and LU 2012/762).

### Baseline measurements

Information regarding smoking habits, alcohol consumption, current medication, leisure-time physical activity and educational level was obtained from the questionnaire. Participants were classified as smokers or non-smokers (including former smokers and never smoker). A daily alcohol intake >40 g for men and >30 g for women was considered to be a high level of alcohol consumption. Physical activity was represented by an overall leisure-time physical activity score calculated by multiplying the duration of specific activities by the corresponding intensity coefficient. Education was categorised as elementary or less, primary, secondary, upper secondary and further education without a degree and with a university degree. Waist circumference (cm) was measured midway between the iliac crest and the lowest rib margin. BP (mmHg) was measured using a mercury-column sphygmomanometer after a 10 min rest in a supine position. Fasting blood glucose levels (mmol/l) were measured from fresh plasma samples collected from the cubital vein after an overnight fast, according to standardised procedures, at the Department of Clinical Chemistry, Skåne University Hospital. LDL-cholesterol levels (mmol/l) were estimated using Friedewald’s formula. Fasting plasma insulin (mU/l) was measured using radioimmunoassay, and values were converted into pmol/l using the conversion factor 6.945. HOMA2-IR was calculated using a HOMA2-IR calculator [24]. C-reactive protein (CRP, mg/l) was analysed using a Tina-quant CRP latex assay (Roche Diagnostics, Switzerland). Renal function, as reflected by eGFR, was estimated from plasma creatinine and cystatin C as previously described [25]. Plasma creatinine was assessed by the Jaffé method using a Beckman Synchron LX20-4 (Beckman-Coulter, USA), and plasma cystatin C was assessed using a particle-enhanced immunonephelometric assay (N Latex Cystatin; Dade Behring, USA). The plasma prostasin concentration was determined using the Proseek Multiplex Oncology I v2 96 × 96 panel (UniProt ID Q16651; Olink Proteomics, Sweden) in blood samples that had been stored at −80°C after collection at baseline. The analyses were performed by SciLifeLab Laboratories (Uppsala, Sweden; www.ScilifeLab.se), and the same batches of reagents were used for sample assays. Olink NPX Manager Software was used for quality control of the samples and technical performance of the assays. During this process, four internal controls were added for all samples, and external controls were used in every analysis. The lower and upper limits of quantification of prostasin were approximately 0.24 and 7800 pg/ml, respectively. The potential impact of haemolysis on prostasin quantification was evaluated by using serial dilutions of haemolysate (0.25–15 mg/ml) in EDTA-treated plasma. The highest concentration of haemolysate (15 mg/ml) had no impact on assay performance. The intra-assay (within a run) and inter-assay (between runs) coefficients of variation were 5% and 19%, respectively. A pre-processing normalisation procedure was used to normalise technical variation within one run and between runs, by subtracting the *C*_q_ value for the extension control and the interplate control for each assay, respectively. The data were set relative to a correction factor, and are expressed as normalised protein expression (NPX) values on a log_2_ scale. The Olink webpage (http://www.olink.se) provides detailed information regarding proteomic panels, proteomics extension assay technology, assay performance, quality control and validation.

### Outcomes

For analyses using diabetes as the endpoint, participants free of diabetes at baseline were followed until incident diabetes, emigration from Sweden, death or the end of follow-up (31 December 2019), whichever came first. Incident cases of diabetes were identified by linkages to both local and national registers, and have been described in detail previously [26]. In brief, information was retrieved from six sources (the Swedish National Diabetes Register, the Regional Diabetes 2000 Register of the Scania Region, the nationwide Swedish drug prescription register, the Swedish inpatient register, the Swedish outpatient register and the Malmö HbA_1c_ Register). In the Swedish National Diabetes Register and the Regional Diabetes 2000 Register, diabetes was diagnosed according to established criteria (fasting plasma glucose concentration ≥7.0 mmol/l with two repeated tests on separate occasions). In the nationwide Swedish drug prescription register, a filled prescription of insulin or glucose-lowering medications (ATC code A10) was required for diagnosis of diabetes. In the Swedish inpatient and outpatient registers, diabetes was diagnosed by a senior physician according to the established criteria. In the Malmö HbA_1c_ Register, individuals were considered to have developed diabetes if they had at least two HbA_1c_ recordings ≥42 mmol/mol (≥6.0%) using the Swedish Mono-S standardisation system (corresponding to 53 mmol/mol [7.0%] according to the US National Glycohemoglobin Standardization Program).

The Swedish cause of death register was used to monitor cause-specific mortality. Cancer mortality was defined as ICD-9 codes 140–239 (http://www.icd9data.com/2007/Volume1/default.htm) or ICD-10 codes C or D00–D48 (http://apps.who.int/classifications/icd10/browse/2016/en), and cardiovascular mortality was defined as ICD-9 codes 390–459 or ICD-10 codes in chapter I, as underlying cause of death.

### Statistical analyses

Baseline characteristics of the study population are presented across quartiles of prostasin, with sex-specific quartile limits. Continuous variables with a skewed distribution are presented as medians (IQR) and were natural-logarithmically transformed before analyses, otherwise results are presented as means ± SD. Categorical variables are presented as numbers and percentages. Differences across quartiles were tested by linear regression for continuous variables and by logistic regression for categorical variables.

The cross-sectional association between prostasin and diabetes was analysed by multivariable logistic regression using three models. Model 1 assessed the crude association. Model 2 was adjusted for age, sex and waist circumference. Model 3 was additionally adjusted for smoking and drinking habits, LDL-cholesterol, systolic BP and anti-hypertensive medication. After excluding prevalent diabetes, the associations between prostasin (as the dependent variable) and baseline fasting blood glucose levels, plasma insulin levels and HOMA2-IR were assessed by multivariable linear regression after adjusting for the above-mentioned confounders. The association between baseline prostasin and incident diabetes was estimated using a Cox proportional hazards regression model, with time-on-study as the timescale. HRs and 95% CIs were calculated. Prostasin was analysed both as a continuous variable (per 1 SD) and grouped variable (quartiles). Potential covariates taken into account were age, sex, waist circumference, smoking and drinking habits, LDL-cholesterol, systolic BP and anti-hypertensive medication. Fasting blood glucose levels or HOMA2-IR were further adjusted for in a separate model, that may be considered as an overadjusted model. Other sensitivity analyses were performed, including additionally adjusting for CRP, eGFR, physical activity and educational level or lipid-lowering drugs, or adjusting for BMI instead of waist circumference in the final model. Possible interactions between prostasin and covariates in relation to diabetes risk were tested by incorporating interaction terms in the multivariable model. The proportional hazard assumption was tested by evaluating the time-dependent effects of prostasin on diabetes risk, and was also visualised using Kaplan–Meier curves. The linearity assumption of the association was examined using restricted cubic spline functions, with knots placed at 20%, 40%, 60% and 80% of prostasin concentration. We also analysed the association between prostasin (both by sex-specific quartiles and by SD) and mortality (all-cause mortality, cardiovascular mortality and cancer mortality) after adjusting for confounders including prior cardiovascular diseases or cancer, where appropriate. Sensitivity analyses were then performed after excluding prior cardiovascular diseases or cancer. The interaction between prostasin and fasting blood glucose levels was evaluated in relation to mortality risk. If an interaction was detected post hoc, analyses were separately performed after dividing participants according to their blood glucose level. Possible effect modifications by competing risk of death were explored using the Fine–Gray proportional sub-distribution hazards models method [27]. C-statistics were calculated to estimate the added predictive value of prostasin to the multivariate model. Statistical significance was accepted with *p* values <0.05 (two-sided). All analyses were performed using SAS version 9.3 for Windows (SAS Institute, USA).

## Results

### Cross-sectional analysis

The study population for the cross-sectional analysis included 4658 participants (age 57.5 ± 5.9 years, 39.9% men), of whom 361 (7.75%) had prevalent diabetes. The distribution plot of prostasin concentration is shown in ESM Fig. 2. ESM Fig. 3 shows the diabetes prevalence in each prostasin quartile. Baseline characteristics for the whole study population across prostasin quartiles are presented in ESM Table 1. The cross-sectional analysis demonstrated a positive association between prostasin and diabetes (ESM Table 2). The multivariable adjusted OR for the highest vs lowest quartile was 1.95 (95% CI 1.39, 2.76; *p* for trend <0.0001). The corresponding OR per 1 SD increase in prostasin was 1.32 (95% CI 1.16, 1.50; *p*<0.0001).

### Longitudinal analysis

After excluding participants with prevalent diabetes, the study population included 4297 participants (age 57.3 ± 5.9 years). The prostasin concentration was 8.44 ± 0.44 NPX for men (*n*=1654) and 8.18 ± 0.48 NPX for women (*n*=2643) (mean ± SD). Baseline characteristics for this group, by quartiles of prostasin, are shown in Table 1. The eGFR values decreased with elevated prostasin levels. Values for all other risk factors showed an increasing trend from the 1st to the 4th quartile of prostasin concentrations. Prostasin levels in individuals without diabetes correlated positively and significantly with fasting blood glucose levels, plasma insulin levels and HOMA2-IR even after adjusting for potential confounders (standardised β coefficients of 0.14, 0.17 and 0.18, respectively; all *p*<0.0001; ESM Table 3).

**Table 1:** Baseline characteristics of the non-diabetic study population (*n*=4297) across sex-specific quartiles (Q1–Q4) of prostasin

	Whole population	Q1 (*n*=1075)	Q2 (*n*=1074)	Q3 (*n*=1075)	Q4 (*n*=1073)	*p* for trend^a^
Prostasin in men (NPX)	8.44 (5.91-9.89)	7.97 (5.91-8.18)	8.33 (8.18-8.46)	8.59 (8.46-8.74)	8.93 (8.74-9.89)	–
Prostasin in women (NPX)	8.18 (6.40-9.72)	7.62 (6.40-7.86)	8.05 (7.87-8.21)	8.35 (8.21-8.50)	8.72 (8.50-9.72)	–
Age (years)	57.3 ± 5.90	56.5 ± 5.90	57.0 ± 5.90	57.8 ± 5.80	57.9 ± 6.00	<0.0001
Men	1654 (38.5)	414 (38.5)	413 (38.5)	414 (38.5)	413 (38.5)	1.00
Waist circumference (cm)	82.5 ± 12.2	80.4 ± 11.6	81.9 ± 11.9	83.1 ± 12.7	84.5 ± 12.3	<0.0001
Smoking	937 (21.8)	112 (10.4)	158 (14.7)	240 (22.3)	427 (39.8)	<0.0001
High alcohol consumption	138 (3.21)	28 (2.60)	30 (2.79)	28 (2.60)	52 (4.85)	0.0070
Systolic BP (mmHg)	140.3 ± 18.7	137.2 ± 18.2	139.2 ± 18.2	141.5 ± 18.6	143.2 ± 19.0	<0.0001
LDL-cholesterol (mmol/l)	4.17 ± 0.98	4.10 ± 0.98	4.09 ± 0.94	4.18 ± 0.97	4.30 ± 1.00	<0.0001
BP-lowering medication	629 (14.6)	115 (10.7)	162 (15.1)	162 (15.1)	190 (17.7)	<0.0001
Fasting blood glucose (mmol/l)	4.89 ± 0.46	4.77 ± 0.42	4.86 ± 0.45	4.92 ± 0.45	5.00 ± 0.47	<0.0001
Plasma insulin (pmol/l) (*n*=4247)	41.7 (27.8-62.5)	34.7 (27.8-48.6)	41.7 (27.8-55.6)	48.6 (27.8-62.5)	48.6 (34.7-69.5)	<0.0001^b^
HOMA2-IR (*n*=4,247)	0.80 (0.50-1.20)	0.70 (0.50-1.00)	0.80 (0.50-1.10)	0.90 (0.55-1.20)	1.00 (0.70-1.40)	<0.0001^b^
CRP (mg/l) (*n*=4205)	1.30 (0.60-2.60)	1.00 (0.60-2.00)	1.20 (0.60-2.30)	1.30 (0.60-2.70)	1.70 (0.80-3.50)	<0.0001^b^
eGFR (ml min^−1^ 1.73 m^−2^) (*n*=4020)	89.0 ± 13.3	90.8 ± 13.2	89.9 ± 12.8	88.3 ± 13.0	87.1 ± 13.8	<0.0001

Values for prostasin are mean (range). Values for insulin, HOMA2-IR and CRP are median (IQR) due to skewed distributions. Values for other continuous variables are means ± SD. Values for categorical variables are *n* (%)

^a^Analysis by linear regression or logistic regression

^b^*p* value for natural log-transformed values of insulin, HOMA2-IR and CRP

Over a mean follow-up period of 21.9 ± 7.0 years, 702 participants developed diabetes. The incident rate was 7.70 per 1000 person-years. The cumulative incidence of diabetes in relation to prostasin quartiles is shown in ESM Fig. 4. Prostasin levels at baseline were higher in those who later developed diabetes. As shown in Table 2, participants with higher prostasin levels had a higher risk of diabetes. After multivariable adjustment in model 3, the HR for diabetes in the highest vs lowest quartile of prostasin was 1.76 (95% CI 1.41, 2.19; *p* for trend <0.0001) and that per 1 SD increase in prostasin was 1.23 (95% CI 1.13, 1.34; *p*<0.0001). The association was attenuated after further adjusting for fasting blood glucose levels or HOMA2-IR in sensitivity analyses, or correcting for competing risk of death, but remained significant. The results were essentially unchanged in all other sensitivity analyses (Table 2). Age, fasting blood glucose and eGFR significantly interacted with prostasin in relation to the risk of diabetes (*p* for interaction =0.028, 0.012 and 0.026, respectively). The association between prostasin and diabetes was more evident among younger participants, in those without impaired fasting blood glucose levels (<5.6 mmol/l, corresponding to a fasting plasma glucose cut-off of 6.1 mmol/l [23]), and in those without impaired renal function (eGFR ≥90 ml min^−1^ [1.73 m]^−2^) (Table 3). The association between prostasin and diabetes was generally linear (*p* for non-linearity =0.30), and there was no evidence that the association was time-dependent (*p* for time dependency =0.49; see also ESM Fig. 4).

**Table 2:** Incidence of diabetes in relation to prostasin by sex-specific quartiles (Q1–Q4) and per 1 SD increase in the non-diabetic population (*n*=4297)

	Prostasin quartiles				*p* for trend^a^	Per 1 SD	*p*^a^
	Q1	Q2	Q3	Q4			
*N* (total=4297)	1075	1074	1075	1073	–	–	–
Incidence (total=702)	131	148	177	246	–	–	–
Incidence (per 1000 person-years)	5.19	6.09	7.61	11.7	–	–	–
HR model 1^b^	Reference	1.18 (0.93, 1.49)	1.48 (1.18, 1.86)	2.38 (1.92, 2.94)	<0.0001	1.41 (1.31, 1.53)	<0.0001
HR model 2^c^	Reference	1.08 (0.85, 1.37)	1.29 (1.03, 1.62)	1.98 (1.60, 2.45)	<0.0001	1.29 (1.19, 1.40)	<0.0001
HR model 3^d^	Reference	1.05 (0.83, 1.34)	1.23 (0.97, 1.54)	1.76 (1.41, 2.19)	<0.0001	1.23 (1.13, 1.34)	<0.0001
Sensitivity analysis							
Replacing waist circumference with BMI	Reference	1.08 (0.85, 1.36)	1.27 (1.01, 1.60)	1.87 (1.51, 2.34)	<0.0001	1.26 (1.16, 1.37)	<0.0001
Additionally adjusted for CRP (*n*=4205)	Reference	1.09 (0.85, 1.39)	1.26 (0.99, 1.59)	1.77 (1.41, 2.22)	<0.0001	1.22 (1.12, 1.33)	<0.0001
Additionally adjusted for baseline eGFR (*n*=4020)	Reference	1.04 (0.82, 1.33)	1.22 (0.96, 1.54)	1.77 (1.41, 2.22)	<0.0001	1.24 (1.13, 1.35)	<0.0001
Additionally adjusted for physical activity and educational level (*n*=4262)	Reference	1.07 (0.84, 1.35)	1.20 (0.95, 1.51)	1.74 (1.39, 2.17)	<0.0001	1.22 (1.12, 1.33)	<0.0001
Additionally adjusted for lipid-lowering drugs	Reference	1.06 (0.83, 1.34)	1.23 (0.97, 1.54)	1.76 (1.41, 2.20)	<0.0001	1.23 (1.13, 1.34)	<0.0001
Overadjusted model							
Additionally adjusted for fasting blood glucose	Reference	0.96 (0.76, 1.22)	1.01 (0.8, 1.28)	1.34 (1.07, 1.68)	0.0043	1.09 (1.00, 1.19)	0.045
Additionally adjusted for HOMA2-IR (*n*=4247)	Reference	1.00 (0.79, 1.27)	1.13 (0.9, 1.42)	1.53 (1.23, 1.92)	<0.0001	1.16 (1.07, 1.27)	0.00060
Accounting for competing risk of death	Reference	1.08 (0.85, 1.36)	1.22 (0.97, 1.53)	1.64 (1.32, 2.05)	<0.0001	1.19 (1.09, 1.30)	<0.0001

Values presented for the models are HR (95% CI)

^a^Analysis by Cox proportional hazards model

^b^Crude model

^c^Adjusted for age, sex and waist circumference

^d^Additionally adjusted for smoking and drinking habits, LDL-cholesterol, systolic BP and anti-hypertensive medication

**Table 3:** The association between prostasin and incident diabetes in subgroups of the population

	Model 1^a^		Model 2^b^	
	Prostasin per 1 SD	*p*^c^	Prostasin per 1 SD	*p*^c^
Age (years)				
<55 (*n*=1534)	1.42 (1.24, 1.62)	<0.0001	1.36 (1.18, 1.57)	<0.0001
55–60 (*n*=1207)	1.33 (1.15, 1.55)	0.00010	1.26 (1.08, 1.47)	0.0027
>60 (*n*=1556)	1.12 (0.97, 1.30)	0.13	1.09 (0.94, 1.26)	0.28
Fasting blood glucose (mmol/l)				
<5.6 (*n*=3920)	1.26 (1.14, 1.38)	<0.0001	1.19 (1.08, 1.31)	0.00060
≥ 5.6 (*n*=377)	1.02 (0.85, 1.22)	0.84	1.01 (0.84, 1.21)	0.90
eGFR (ml/min per 1.73 m^2^)				
≥ 90 (*n*=1938)	1.45 (1.28, 1.64)	<0.0001	1.36 (1.20, 1.54)	<0.0001
<90 (*n*=2082)	1.19 (1.05, 1.33)	0.0051	1.13 (1.00, 1.27)	0.059

Values presented for the models are HR (95% CI)

^a^Adjusted for age, sex and waist circumference

^b^Additionally adjusted for smoking and drinking habits, LDL-cholesterol, systolic BP and anti-hypertensive medication (excluding the stratification variable)

^c^Analysis by Cox proportional hazards model

Over a mean follow-up period of 23.5 ± 6.1 years, 651 participants died from cancer. Prostasin was significantly associated with cancer mortality. The cumulative cancer mortality in relation to prostasin quartiles is illustrated in ESM Fig. 5. After multivariable adjustment, the HR was 1.43 (95% CI 1.14,1.80) comparing the highest vs lowest quartile of prostasin (*p* for trend =0.00080) and that per 1 SD increase in prostasin was 1.13 (95% CI 1.04, 1.23; *p*=0.0058) (Table 4). The results remained substantially unchanged after excluding cancer at baseline. Additional accounting for competing risk of death slightly attenuated the association (Table 4). A significant interaction was found between prostasin and fasting blood glucose for risk of cancer mortality (*p* for interaction =0.022). The HR for cancer mortality were 1.52 (95% CI 1.07, 2.16; *p*=0.019) and 1.11 (95% CI 1.01, 1.21; *p*=0.025) per 1 SD increase in prostasin, respectively, among participants with (*n*=377) and without (*n*=3920) impaired fasting blood glucose levels at baseline (data not shown).

**Table 4:** Incidence of cancer mortality in relation to prostasin by sex-specific quartiles (Q1–Q4) and per 1 SD increase in the non-diabetic population (*n* = 4297)

	Prostasin quartiles				*p* for trend ^a^	Per 1 SD	*p* ^a^
	Q1	Q2	Q3	Q4			
*N* (total = 4297)	1075	1074	1075	1073	–	–	–
Incidence (total = 651)	129	138	166	218	–	–	–
Incidence (per 1000 person-years)	4.88	5.33	6.63	9.27	–	–	–
Model 1^b^	Reference	1.05 (0.83, 1.34)	1.28 (1.02, 1.62)	1.80 (1.44, 2.25)	<0.0001	1.26 (1.16, 1.37)	<0.0001
Model 2^c^	Reference	1.01 (0.80, 1.29)	1.15 (0.91, 1.45)	1.42 (1.13, 1.78)	0.0010	1.13 (1.04, 1.23)	0.0058
Model 3^d^	Reference	1.02 (0.80, 1.30)	1.16 (0.92, 1.46)	1.43 (1.14, 1.80)	0.00080	1.13 (1.04, 1.23)	0.0058
Sensitivity analysis							
Excluding prior cancer (*n* = 4065)	Reference	1.03 (0.80, 1.33)	1.17 (0.91, 1.50)	1.43 (1.12, 1.82)	0.0016	1.13 (1.03, 1.24)	0.010
Accounting for competing risk of death	Reference	1.01 (0.80, 1.29)	1.14 (0.90, 1.44)	1.34 (1.07, 1.69)	0.0066	1.10 (1.00, 1.21)	0.040

Values presented for the models are HR (95% CI)

^a^Analysis by Cox proportional hazards model

^b^Adjusted for age, sex and waist circumference

^c^Additionally adjusted for smoking and drinking habits, LDL-cholesterol, systolic BP and anti-hypertensive medication

^d^Additionally adjusted for prior diagnosis of cancer

Prostasin did not significantly improve the predictive ability for incident diabetes or cancer mortality when added to the multivariable model (*p* for change in C-statistic =0.29 and 0.36, respectively) (data not shown). In addition, prostasin was found to be significantly and linearly associated with all-cause mortality (*p* for interaction =0.16), but no significant association was found for cardiovascular mortality (ESM Table 4).

## Discussion

In this study, we have demonstrated that prostasin was positively associated with the development of diabetes, independently of traditional risk factors. We also found that prostasin was associated with increased cancer mortality risk and total mortality, and the strength of the association between prostasin and cancer mortality was much stronger in participants with elevated baseline blood glucose levels. In contrast, there was no significant relationship between prostasin and cardiovascular mortality.

Prostasin is usually described as a glycosylphos-phatidylinositol-anchored protein, but it also circulates in blood [10, 11, 16, 17, 28], and the circulating concentration correlates with the local expression levels [10, 16, 17]. In a study by Sekine et al, serum prostasin levels were observed to be lower in individuals with diabetes than those without [17]. However, the sample size was much smaller than in our study (*n*=39–40 per group), and there was no control for confounding in that study, which may explain why the results were different. To our knowledge, no previous evidence has been provided regarding the association between prostasin levels and the incidence of diabetes. The present results were generated from a large study population and with potential confounding factors taken into account. Moreover, the positive cross-sectional associations were confirmed by the prospective analyses. We have therefore addressed this knowledge gap by showing that plasma prostasin is positively associated with diabetes.

Our current understanding of the role of prostasin in glucose metabolism comes from two experimental studies [16, 17]. The study by Uchimura et al suggested a protective role of prostasin in glucose metabolism due to its anti-inflammatory effect [16]. Liver is an important source of circulating prostasin [16, 17], as well as a major site of insulin resistance in diabetes [29]. Uchimura et al found that prostasin promoted hepatic insulin sensitivity via modification of Toll-like receptor 4 (TLR4) signalling [16]. TLR4 is a key promoter of inflammatory responses [30, 31]. An anti-inflammatory effect of prostasin overexpression by downregulating TLR4 expression has been observed against colitis in the colonic epithelium [32]. The ability to suppress Sphk1/S1p/Stat3/Akt inflammatory signalling has also been proposed as a potential mechanism of prostasin action against inflammation [8]. Apart from its role in inflammation, prostasin may also amplify phosphorylation of extracellular signal-regulated kinase through the prostasin/epidermal growth factor receptor/extracellular signal-regulated kinase axis in the liver, thus improving glucose tolerance and hepatic steatosis [17].

There are several other possible explanations for the association between prostasin and diabetes that have not yet been tested. Recently, α-ENaC knockout in a murine pancreatic beta cell line has been found to increase cell viability and insulin content [20]. We assume that prostasin, as a strong stimulator of ENaCs by cleaving the γ subunit [1, 3], may also influence pancreas function. The link between prostasin and diabetes may also be related to Wnt/β-catenin signalling. Increased prostasin expression in the intestinal epithelium has been found to inhibit Wnt/β-catenin signalling [15], which has a pivotal role in regulating insulin synthesis and secretion, and pancreatic beta cell proliferation [33]. Further studies are needed to test these assumptions.

A recent Mendelian randomisation study by Pietzner et al suggested that *PRSS8*, a gene encoding prostasin, was causally associated with Alzheimer’s disease [34], possibly related to TLR4-mediated neuroinflammation [35]. Current evidence also supports potential protective mechanisms of prostasin against hyperglycaemia [16, 17], including that mediated by TLR4 [16]. However, whereas Pietzner et al consistently observed a negative association between plasma prostasin and risk of Alzheimer’s disease, we found a positive association between prostasin and diabetes. This association was greatly attenuated by adjusting for baseline fasting blood glucose levels. It is thus hypothesised that elevated plasma prostasin levels may be a compensatory response to hyperglycaemia but may be insufficient to stop or reverse the deterioration of glucose control. Further Mendelian randomisation analysis may help to confirm the causality between prostasin and diabetes. The predictive value of prostasin for incident diabetes was better in younger participants, or those with lower blood glucose levels or better renal function. Because prostasin may be secreted into urine [36], normal kidney function may help to maintain optimal plasma prostasin levels.

In this study, plasma prostasin levels were associated with cancer mortality in the general population. This association has not been investigated previously. Serum prostasin concentration has been proposed as a prognostic marker for ovarian cancer [10, 11] but information is lacking regarding other types of cancer. The role of prostasin in tumour biology has been suggested to be tissue-dependent, and it is hard to identify here the exact explanation for the observed association. Nevertheless, it is interesting that the association was found to be much stronger in participants with higher baseline blood glucose concentrations. This observation sheds some light into the known diabetes and cancer connection [12–14]. Several diabetes-associated biological pathways, such as inflammation, endoplasmic reticulum stress, the epithelial-to-mesenchymal transition, Akt signalling and Wnt/β-catenin signalling, also participate in carcinogenesis, invasion or metastasis [13, 14, 33, 37]. Prostasin has a role in regulating these pathways [8, 15, 16]. Therefore, prostasin may potentially mediate the process from hyperglycaemia to cancer, or at least may act as a marker for cancer susceptibility in participants with hyperglycaemia. If a casual association were established in the future, prostasin may then be considered as a therapeutic target for treating both diabetes and cancer.

The strengths of our study include the large sample size, the prospective design and the long-term follow-up. However, there are several limitations to this study. One is that prostasin levels were measured in arbitrary units (NPX values), and thus could not be compared directly with absolute values. However, since the study cohort was taken from the general population, the overall prostasin levels in this study may be regarded as normal by definition. The prostasin concentration was measured using a Proseek Multiplex Oncology panel. This method uses paired DNA-labelled antibodies and has high specificity. We did not make any Bonferroni adjustments for biomarkers not analysed in this study. However, even with a conservative Bonferroni approach, the relationships between prostasin and incidence of diabetes would still be statistically significant. Prostasin levels were measured using frozen blood samples stored at −80°C for over a decade, and its stability during long-term storage is unclear. Nevertheless, the fact that a predictive ability of prostasin was observed in this study suggests that the analytes are sufficiently stable to preserve clinical signals, even though the identified values may not be exactly the same as if measured in fresh samples. In addition, serial measurements of prostasin and risk factors over time are lacking in this study. Another limitation is that diabetes types were not determined in this study. As all the participants in cohort analysis were middle-aged adults without pre-existing diabetes, it is very likely that the incident cases were type 2 diabetes. Incident diabetes cases were identified by multiple registers [26], which included patients receiving glucose-lowering drug treatment as well as those with dietary treatment only. However, it is still possible that some cases have been missed. Furthermore, diabetes may remain undetected and undiagnosed for a long time. Due to the observational design, our findings are descriptive in nature. Further studies are warranted to trace the exact origins of prostasin in blood, and to determine whether the association between prostasin and diabetes is causal.

In conclusion, plasma prostasin is a new potential risk marker for development of diabetes and for cancer mortality risk, especially in individuals with high blood glucose levels, which may shed new light on the relationship between diabetes and cancer.

## Supplementary information

ESM (PDF 515 kb)

## Data Availability

The data that support the findings of this study are available from Lund University, but restrictions apply to the availability of these data, which were used under license for the current study, and so are not publicly available. However, data may be available from the corresponding author (GE) upon reasonable request and with permission of Lund University.

## Abbreviations

CRP: C-reactive protein
ENaC: Epithelial sodium channel
NPX: Normalised protein expression
TLR4: Toll-like receptor 4
