# Supplementary material for: Plasma prostasin: a novel risk marker for incidence of diabetes and cancer mortality
Source: Diabetologia. 2022 Aug 4;65(10):1642–51. doi: 10.1007/s00125-022-05771-w (PMC9477896; doi:10.1007/s00125-022-05771-w)
Supplement: Supplementary file 1 — (PDF 515 kb) [file 125_2022_5771_MOESM1_ESM.pdf]

Electronic Supplementary Material (ESM)

Plasma prostasin: a novel risk marker for incidence of diabetes and cancer mortality

Xue Bao[1,2], Biao Xu[1], Iram Faqir Muhammad[2], Peter M. Nilsson[2], Jan Nilsson[2] and Gunnar Engström[2]

[1]Department of Cardiology, Nanjing Drum Tower Hospital, The Affiliated Hospital of Nanjing University Medical School, Nanjing, China

[2]Department of Clinical Sciences, Malmö, Lund University, Malmö, Sweden

Corresponding authors:

Biao Xu xubiao62@nju.edu.cn

Gunnar Engström gunnar.engstrom@med.lu.se

ORCID identifiers:

Xue Bao: 0000-0003-3777-5988

Biao Xu: 0000-0003-3404-8582

Gunnar Engström: 0000-0002-8618-9152

ESM Table 1 Baseline characteristics of the study population (n = 4658) across sex-specific quartiles (Q1–Q4) of prostasin

	Whole population	Q1 (n = 1165)	Q2 (n = 1164)	Q3 (n = 1165)	Q4 (n = 1164)	p for trend[a]
Prostasin in men (NPX)	8.46 (5.91-9.89)	7.89 (5.91-8.19)	8.35 (8.19-8.48)	8.61 (8.48-8.76)	8.99 (8.76-9.89)	–
Prostasin in women (NPX)	8.19 (6.40-9.78)	7.89 (5.91-8.19)	8.35 (8.19-8.48)	8.61 (8.48-8.76)	8.99 (8.76-9.89)	–
Diabetes	361 (7.75)	57 (4.89)	64 (5.50)	90 (7.73)	150 (12.89)	<0.0001
Age (years)	57.5 ±5.90	56.6 ±5.90	57.2 ±5.90	57.9 ±5.90	58.2 ±6.00	<0.0001
Men	1858 (39.9)	465 (39.9)	464 (39.9)	465 (39.9)	464 (39.9)	0.99
Waist circumference (cm)	83.4 ±12.8	81.1 ±12.0	82.5 ±12.2	84.1 ±13.5	86 ±12.9	<0.0001
Smoking	1007 (21.6)	119 (10.2)	178 (15.3)	264 (22.7)	446 (38.3)	<0.0001
High alcohol consumption	160 (3.43)	32 (2.75)	31 (2.66)	32 (2.75)	65 (5.58)	0.0004
Systolic BP (mmHg)	140.9 ±18.9	137.8 ±18.4	139.6 ±18.3	142.1 ±18.7	144.3 ±19.5	<0.0001
LDL-cholesterol (mmol/l)	4.17 ±0.98	4.10 ±0.99	4.12 ±0.96	4.17 ±0.95	4.29 ±1.02	<0.0001
BP-lowering medication	744 (16.0)	133 (11.4)	181 (15.6)	196 (16.8)	234 (20.1)	<0.0001
Fasting blood glucose (mmol/l)	4.90 (4.60-5.30)	4.80 (4.50-5.10)	4.80 (4.60-5.20)	4.90 (4.60-5.30)	5.10 (4.70-5.50)	<0.0001[b]
Plasma insulin (pmol/l) (n = 4627)	41.7 (27.8-62.5)	34.7 (27.8-48.6)	41.7 (27.8-55.6)	48.6 (27.8-62.5)	55.6 (34.7-76.4)	<0.0001[b]
HOMA2-IR (n = 4627)	0.80 (0.50-1.20)	0.70 (0.50-10.0)	0.80 (0.50-1.10)	0.90 (0.60-1.30)	1.10 (0.70-1.60)	<0.0001[b]
CRP (mg/l) (n = 4556)	1.30 (0.70-2.70)	1.00 (0.60-2.10)	1.20 (0.60-2.50)	1.40 (0.70-2.80)	1.70 (0.80-3.70)	<0.0001[b]
eGFR (ml min^{-1} 1.73 m^{-2}) (n = 4351)	89.0 ±13.5	90.8 ±13.4	89.7 ±13.0	88.4 ±13.3	87.1 ±14.1	<0.0001

CRP, C-reactive protein; HOMA2-IR, Homeostasis Model Assessment-2 Insulin Resistance; NPX, Normalised protein expression. Values for prostasin are mean (range). Values for fasting glucose, insulin, HOMA2-IR and CRP are median (IQR) due to skewed distributions. Values for other continuous variables are means ± SD. Values for categorical variables are n (%)

[a]Analysis by linear regression or logistic regression

[b]p value for natural log-transformed values of fasting glucose, insulin, HOMA2-IR and CRP

ESM Table 2 Prevalence of diabetes in relation to prostasin by sex-specific quartiles (Q1–Q4) and per 1 SD increase (n = 4658)

	Q1 (n = 1165)	Q2 (n = 1164)	Q3 (n = 1165)	Q4 (n = 1164)	p for trend [a]	Per 1 SD	p[a]
No. of diabetes (n = 361)	57	64	90	150	–	–	–
OR Model 1[b]	Reference	1.17 (0.81, 1.69)	1.66 (1.18, 2.35)	2.92 (2.14, 4.05)	<0.0001	1.67 (1.48, 1.87)	<0.0001
OR Model 2[c]	Reference	1.04 (0.71, 1.52)	1.24 (0.87, 1.79)	2.02 (1.45, 2.84)	<0.0001	1.34 (1.18, 1.52)	<0.0001
OR Model 3[d]	Reference	1.03 (0.70, 1.50)	1.21 (0.84, 1.74)	1.95 (1.39, 2.76)	<0.0001	1.32 (1.16, 1.50)	<0.0001

[a]Analysis by Multiple logistic regression. Values presented for the models are OR (95% CI)

[b]Crude model

[c]Adjusted for age, sex and waist circumference

[d]Additionally adjusted for smoking and drinking habits, LDL-cholesterol, systolic blood pressure, and anti-hypertensive drug medication

ESM Table 3 Associations between prostasin and fasting blood glucose, plasma insulin and HOMA2-IR at baseline in individuals without diabetes

	Standardized β coefficient	Standard error	p[a]
**Model 1**[b]			
Fasting blood glucose (n = 4297)	0.25	0.016	<0.0001
Plasma insulin (n = 4247)	0.23	0.014	<0.0001
HOMA2-IR (n = 4247)	0.24	0.013	<0.0001
**Model 2**[c]			
Fasting blood glucose (n = 4297)	0.17	0.016	<0.0001
Plasma insulin (n = 4247)	0.17	0.015	<0.0001
HOMA2-IR (n = 4247)	0.18	0.015	<0.0001
**Model 3**[d]			
Fasting blood glucose (n = 4297)	0.14	0.016	<0.0001
Plasma insulin (n = 4247)	0.17	0.015	<0.0001
HOMA2-IR (n = 4247)	0.18	0.015	<0.0001

HOMA2-IR, Homeostasis Model Assessment-2 Insulin Resistance

[a]Analysis by multivariable linear regression with prostasin as the dependent variable. Insulin and HOMA2-IR were natural-logarithmically transformed before analyses

[b]Crude model

[c]Adjusted for age, sex and waist circumference

[d]Additionally adjusted for smoking and drinking habits, LDL-cholesterol, systolic blood pressure, and anti-hypertensive medication

ESM Table 4 Incidence of all-cause mortality and cardiovascular mortality in relation to prostasin by sex-specific quartiles (Q1–Q4) and per 1 SD increase (*n* = 4297)

	Q1 (*n* = 1075)	Q2 (*n* = 1074)	Q3 (*n* = 1075)	Q4 (*n* = 1073)	*p* for trend[a]	Per 1 SD	*p*[a]
**All-cause mortality**							
Incidence	354	386	434	558	–	–	–
Incidence per 1000 person years	13.4	14.9	17.3	23.7	–	–	–
Model 1[b]	Reference	1.07 (0.92, 1.23)	1.18 (1.02, 1.36)	1.63 (1.43, 1.87)	<0.0001	1.23 (1.16, 1.29)	<0.0001
Model 2[c]	Reference	1.04 (0.90, 1.20)	1.07 (0.93, 1.23)	1.33 (1.16, 1.53)	<0.0001	1.12 (1.06, 1.18)	<0.0001
**Cardiovascular mortality**							
Incidence	108	108	124	156	–	–	–
Incidence per 1000 person years	4.08	4.17	4.95	6.63	–	–	–
Model 1[b]	Reference	0.92 (0.71, 1.21)	1.00 (0.77, 1.30)	1.35 (1.05, 1.73)	0.0098	1.13 (1.02, 1.24)	0.018
Model 2[c]	Reference	0.90 (0.69, 1.17)	0.90 (0.70, 1.17)	1.10 (0.85, 1.42)	0.39	1.03 (0.93, 1.14)	0.59
Model 3[d]	Reference	0.90 (0.69, 1.18)	0.89 (0.68, 1.15)	1.06 (0.82, 1.37)	0.59	1.01 (0.92, 1.12)	0.84
Sensitivity analysis							
Excluding prior cardiovascular events (*n* = 4204)	Reference	0.88 (0.66, 1.16)	0.92 (0.70, 1.2)	1.11 (0.85, 1.44)	0.34	1.03 (0.93, 1.14)	0.59
Accounting for competing risk of death	Reference	0.88 (0.68, 1.15)	0.85 (0.65, 1.11)	0.94 (0.73, 1.21)	0.70	0.97 (0.87, 1.07)	0.51

[a]Analysis by Cox proportional hazards model. Values presented for the models are HR (95% CI)

[b]Adjusted for age, sex and waist circumference

[c]Additionally adjusted for smoking and drinking habits, LDL-cholesterol, systolic BP, and anti-hypertensive medication

[d]Additionally adjusted for prior cardiovascular events

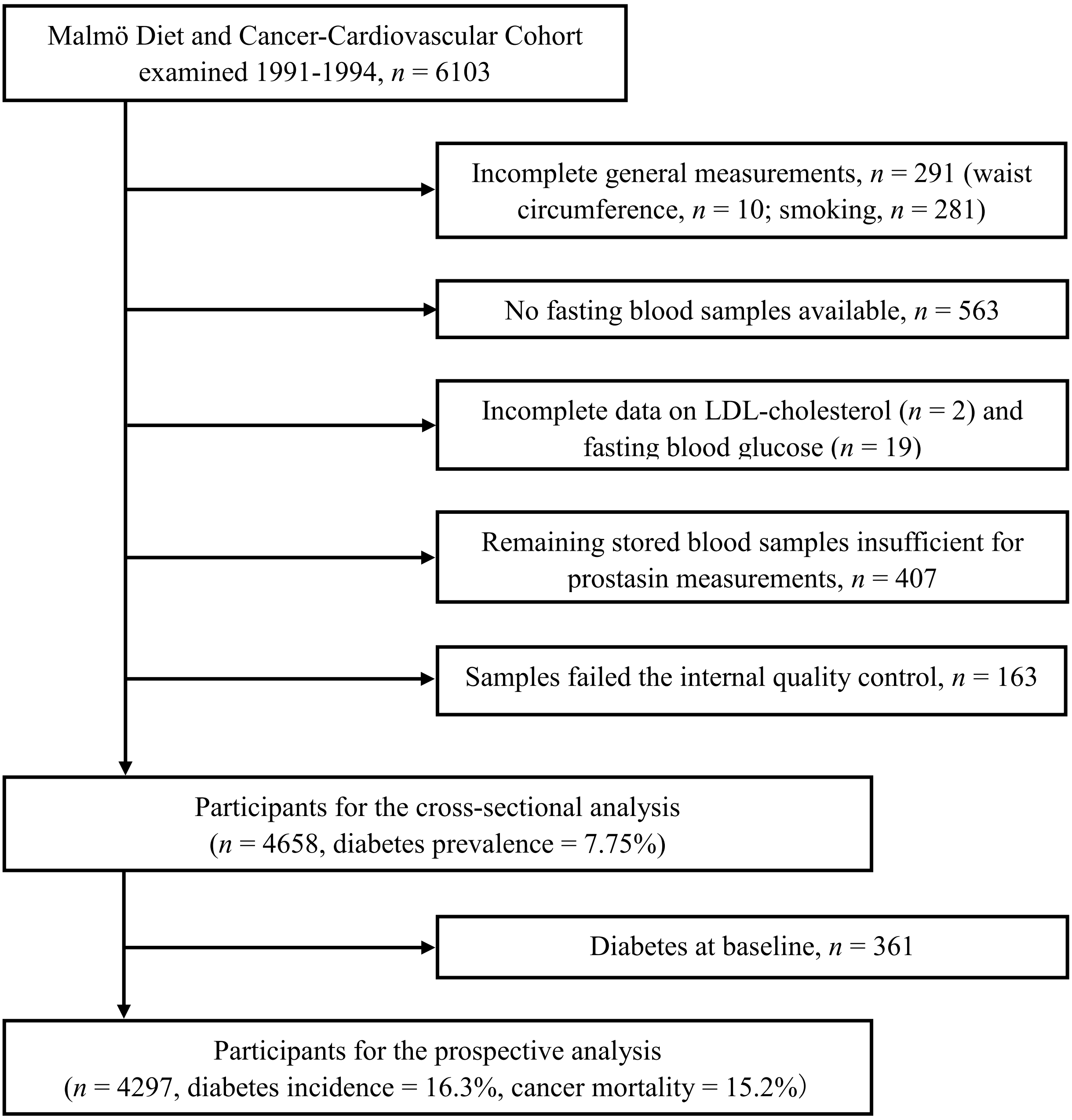

ESM Fig. 1 Study population flow chart

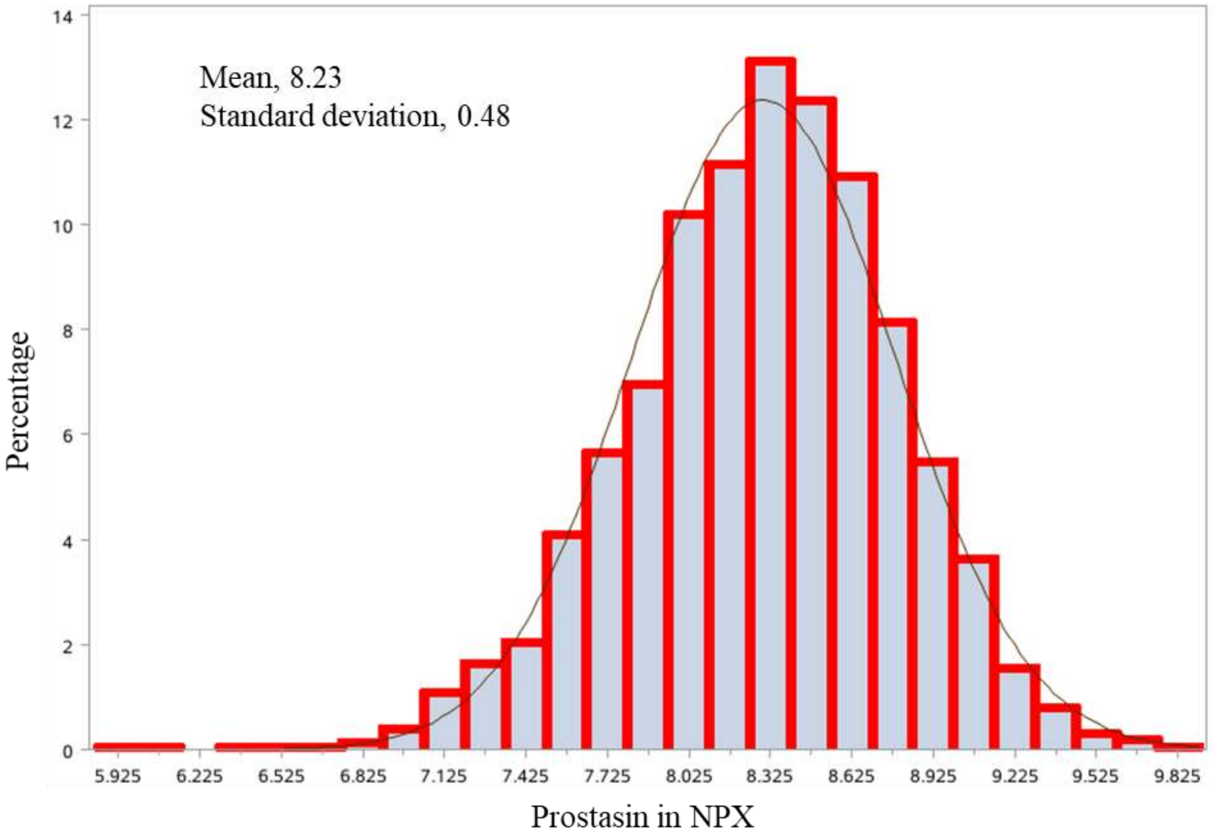

ESM Fig. 2 Distribution plot of prostasin

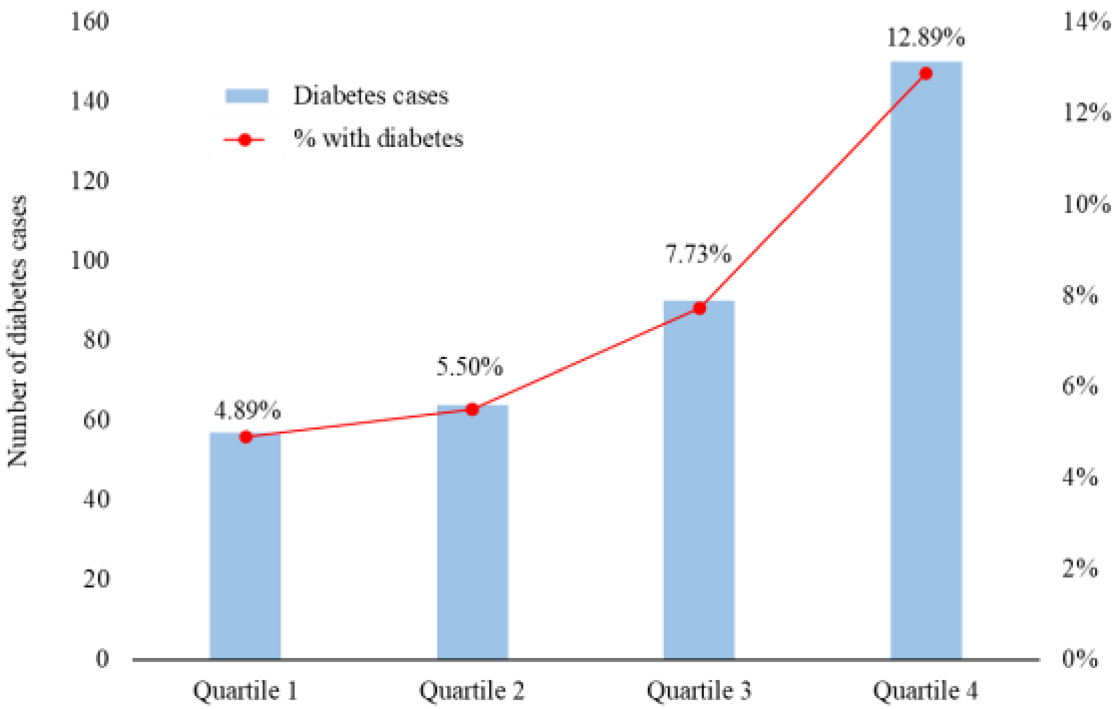

ESM Fig. 3 Number of prevalent diabetes cases and the percentage (%) of people with diabetes in each quartile of prostasin

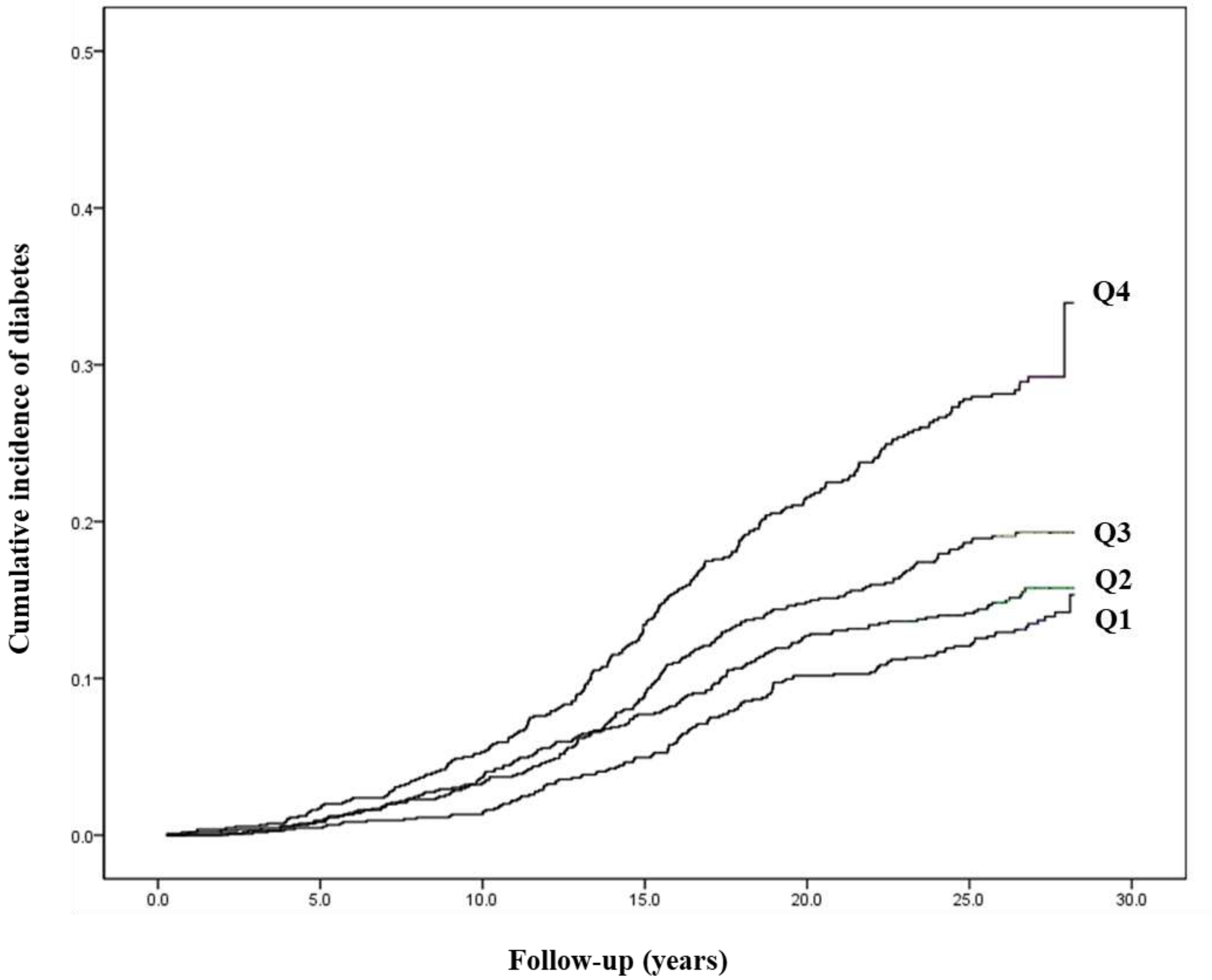

ESM Fig. 4 Cumulative incidence of diabetes in relation to prostasin quartiles (Q1–Q4)

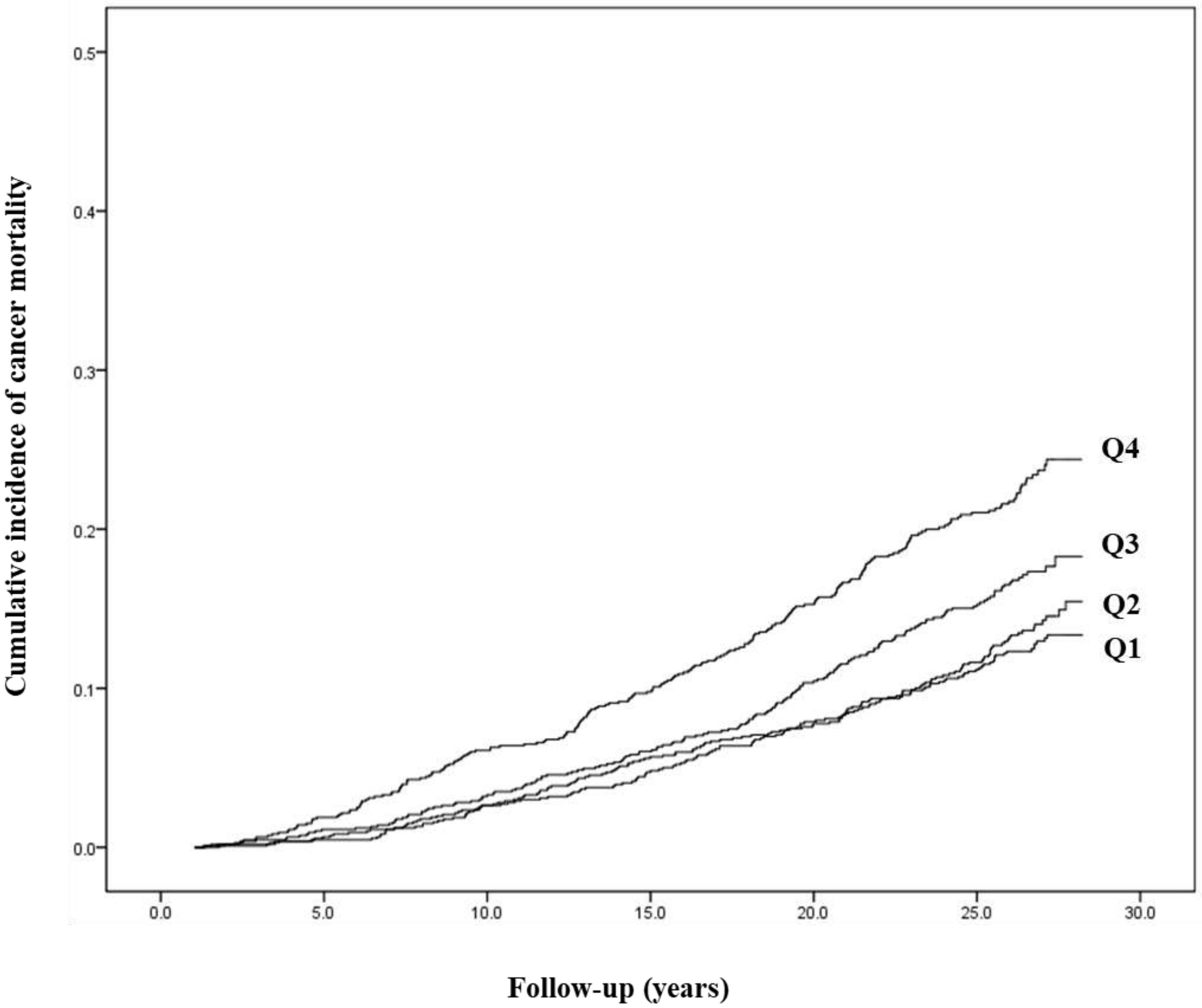

ESM Fig. 5 Cumulative cancer mortality in relation to prostasin quartiles (Q1–Q4)
